# Challenges and Potential of Remote Sensing for Assessing *Salmonella* Risk in Water Sources: Evidence from Chile

**DOI:** 10.3390/microorganisms13071539

**Published:** 2025-06-30

**Authors:** Rayana Santos Araujo Palharini, Makarena Sofia Gonzalez Reyes, Felipe Ferreira Monteiro, Lourdes Milagros Mendoza Villavicencio, Aiko D. Adell, Magaly Toro, Andrea I. Moreno-Switt, Eduardo A. Undurraga

**Affiliations:** 1 Departamento de Prevención de Riesgos y Medio Ambiente, Universidad Tecnológica Metropolitana, Santiago 8330383, Chile; 2 Center for Bioinformatics and Integrative Biology, Facultad de Ciencias de la Vida, Universidad Andrés Bello, Santiago 8370186, Chile; makarenagonzalezreyes@gmail.com; 3 Centro de Tecnologia, Departamento de Transportes e Geomatica, Campus Ministro Petrônio Portella, Bairro Ininga, Teresina 64049-550, Brazil; felipefmonteiro@gmail.com; 4 Departamento de Biometría e Estatística Aplicada, Universidade Federal Rural de Pernambuco, Recife 52171-900, Brazil; lumimevi@gmail.com; 5 Escuela de Medicina Veterinaria, Facultad de Ciencias de La Vida, Universidad Andrés Bello, Santiago 8370035, Chile; aiko.adell@unab.cl; 6 Joint Institute for Food Safety and Applied Nutrition (JIFSAN), University of Maryland, College Park, MD 20740, USA; mtoroiba@umd.edu; 7 Escuela de Medicina Veterinaria, Pontificia Universidad Católica de Chile, Santiago 7820436, Chile; andrea.moreno@uc.cl; 8 Escuela de Gobierno, Pontificia Universidad Católica de Chile, Santiago 7820436, Chile; eundurra@uc.cl; 9 Research Center for Integrated Disaster Risk Management (CIGIDEN), Santiago 7820436, Chile

**Keywords:** Chile, climate change, waterborne diseases, salmonella, remote sensing

## Abstract

Waterborne illnesses, including those caused by *Salmonella*, are an increasing public health challenge, particularly in developing countries. Potential sources of salmonellosis include fruits and vegetables irrigated/treated with surface water, leading to human infections. *Salmonella* causes millions of gastroenteritis cases annually, but early detection through routine water quality surveillance is time-consuming, requires specialized equipment, and faces limitations, such as coverage gaps, delayed data, and poor accessibility. Climate change-driven extreme events such as floods and droughts further exacerbate variability in water quality. In this context, remote sensing offers an efficient and cost-effective alternative for environmental monitoring. This study evaluated the potential of Sentinel-2 satellite imagery to predict *Salmonella* occurrence in the Maipo and Mapocho river basins (Chile) by integrating spectral, microbiological, climatic, and land use variables. A total of 1851 water samples collected between 2019 and 2023, including 704 positive samples for *Salmonella*, were used to develop a predictive model. Predicting *Salmonella* in surface waters using remote sensing is challenging for several reasons. Satellite sensors capture environmental proxies (e.g., vegetation cover, surface moisture, and turbidity) but not pathogens. Our goal was to identify proxies that reliably correlate with *Salmonella*. Twelve spectral indices (e.g., NDVI, NDWI, and MNDWI) were used as predictors to develop a predictive model for the presence of the pathogen, which achieved 59.2% accuracy. By spatially interpolating the occurrences, it was possible to identify areas with the greatest potential for *Salmonella* presence. NDWI and AWEI were most strongly correlated with *Salmonella* presence in high-humidity areas, and spatial interpolation identified the higher-risk zones. These findings reveal the challenges of using remote sensing to identify environmental conditions conducive to the presence of pathogens in surface waters. This study highlights the methodological challenges that must be addressed to make satellite-based surveillance an accessible and effective public health tool. By integrating satellite data with environmental and microbiological analyses, this approach can potentially strengthen low-cost, proactive environmental monitoring for public health decision-making in the context of climate change.

## 1. Introduction

Foodborne diseases represent a major global public health concern, affecting both industrialized and developing countries [1]. One of the main contributing factors to their spread is the use of contaminated water during various stages of agricultural production, including the irrigation, washing, and processing of food products [2,3,4]. This water can serve as an indirect vehicle for the transmission of various enteric pathogens, including *Cryptosporidium*, *Giardia*, *Campylobacter*, *Escherichia coli*, *Shigella*, and *Salmonella* [5].

Water contamination may originate from multiple sources, including untreated wastewater discharges, agricultural runoff containing manure and fertilizers, and improper disposal of industrial waste [2,6]. These practices not only compromise the quality of water bodies used for human consumption, agricultural irrigation, and recreational activities, but also pose a significant public health risk by facilitating the entry of pathogenic microorganisms into the food chain [7].

Globally, *Salmonella enterica* is responsible for approximately 93.8 million cases of gastroenteritis and 155,000 deaths annually [8]. The risk of infection increases significantly in areas with poor sanitation, where this bacterium contributes substantially to the burden of diarrheal diseases, primarily affecting children and the elderly.

In Chile, *Salmonella* has a growing impact on public health. Between 2014 and 2018, 10,104 cases of salmonellosis were reported [9], with an increasing incidence over time. The Metropolitan Region has been particularly relevant in epidemiological terms: during the same period, 43.8% of the *Salmonella* strains confirmed nationwide originated from this region [9].

The Metropolitan Region of Santiago concentrates nearly 40% of the national population, with more than 7.4 million inhabitants [10], exerting significant pressure on its water resources exerting significant pressure on its water resources. The Maipo River Basin, which spans over 15,000 km^2^ [11], supplies around 70% of the current drinking water demand and approximately 90% of the irrigation demand in the region, serving agricultural areas [11]. One of the main tributaries of this basin is the Mapocho River, which drains an area of approximately 4230 km^2^ in the northern sector of the valley [12], supplying drinking water to the eastern districts of Santiago [13]. Both the Mapocho and Maipo rivers have been the focus of recent studies aimed at evaluating the presence of *Salmonella* in surface water [14]. These investigations confirmed the detection of the pathogen in both basins, with 31.7% of the samples testing positive in the Mapocho River and 25.8% in the Maipo River [14].

In this context, characterized by high population density, intensive agricultural activity, and diffuse pollution sources, effective tools for the detection and monitoring of pathogens in surface water bodies are critical. However, the early detection of bacteria, such as *Salmonella*, remains a challenge due to their intermittent presence and the limitations of traditional microbiological methods. Although accurate, these methods are often costly, time-consuming, and require specialized infrastructure [15], hindering their systematic and real-time application, especially in remote or resource-limited settings.

Against this backdrop, satellite remote sensing has emerged as a promising tool for monitoring water quality. This technology enables continuous, large-scale, and cost-effective monitoring of various physicochemical and biological parameters that serve as indirect indicators of contamination [16]. While satellite sensors cannot directly detect bacteria, they can identify changes in the optical properties of water, such as color, turbidity, dissolved organic matter, or chlorophyll, which are associated with conditions favorable for bacterial proliferation [17].

Studies have demonstrated the potential to predict the presence of bacteria, such as *Vibrio* [18] and *Enterococcus* [19], using models that integrate environmental variables derived from remote sensing, including surface water temperature, phytoplankton concentration, and spectral water characteristics [16,17,20]. In the case of *Salmonella*, although direct detection through remote sensing is not yet feasible, monitoring environmental variables associated with its occurrence could provide an effective alternative for developing early warning systems.

To further improve the accuracy and applicability of predictive models, it is essential to incorporate non-optical and non-remote-sensing variables. These include urban population density [21], land use (agricultural, industrial, or residential) [22], proximity to wastewater treatment plants, and degree of soil erosion within watersheds [23]. Although not directly observable by satellite sensors, these factors significantly influence contamination dynamics and can be obtained from cadastral records and census data [24,25,26]. Integrating such variables into remote sensing-based models could enable a more comprehensive assessment of microbiological risks in surface waters.

The integration of remote sensing data with environmental, geographic, and socioeconomic information thus offers a multidimensional approach for anticipating risk scenarios and informing more effective mitigation strategies. Therefore, this study aimed to evaluate the potential of remote sensing as a tool for predicting the presence of *Salmonella* in surface waters from the Maipo and Mapocho river basins in central Chile. To this end, high-resolution Sentinel-2 satellite imagery was combined with the presence/absence data of the pathogen in water samples, applying spectral indices and spatial interpolation models to identify areas with a higher probability of contamination and to analyze their relationship with environmental factors. This approach aims to support the development of more efficient and prevention-oriented environmental surveillance systems.

## 2. Materials and Methods

### 2.1. Study Area Description

**Figure 1:**
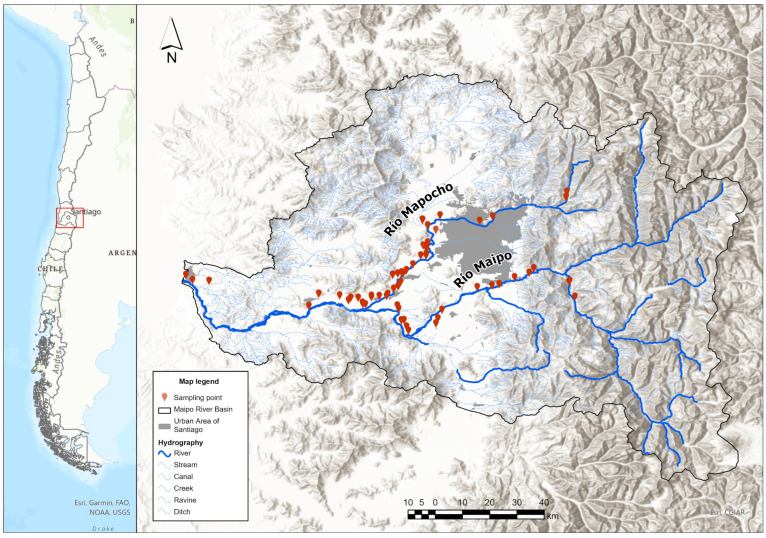
Map of the study area in the Metropolitan Region of Santiago, Chile. The red markers indicate the sampling points from which water was collected for microbiological analysis. The Mapocho and Maipo rivers are outlined in blue, representing the main watercourses evaluated in this study.

The data used in this study cover the Maipo and Mapocho river basins, which are located in the Metropolitan Region of Santiago, Chile (Figure 1). These basins represent a strategic setting for evaluating the relationship between environmental factors and microbiological contamination, as they include urban, peri-urban, and agricultural areas and provide water for both crop irrigation and human consumption.

The Metropolitan Region of Santiago is home to more than 7.1 million people, accounting for approximately 40% of the national population, with a population density of 461.77 inhabitants per square kilometer [27,28]. This high population density, combined with agricultural and industrial development, places significant pressure on the local water resources.

The Maipo River Basin, which spans over 15,000 km^2^, supplies approximately 70% of the region’s drinking water and around 90% of its irrigation needs, serving agricultural areas [11]. One of its main tributaries is the Mapocho River, which drains an area of approximately 4230 km^2^ [12] in the northern part of the valley and supplies drinking water to the eastern districts of Santiago [13].

Both river basins have been the focus of recent studies documenting the presence of *Salmonella* in surface waters. These studies, which focused on microbiological risk assessment, reported detection rates of 31.7% in the Mapocho River and 25.8% in the Maipo River [14].

### 2.2. Source of Data on Salmonella Occurrence

The *Salmonella* incidence data used in this study were obtained from the work published by Toro et al. (2022) [14], who conducted a sampling campaign between April 2019 and February 2020 in the Maipo and Mapocho river basins in central Chile. During this period, 540 surface water samples were collected from 30 georeferenced sampling sites covering various water bodies, including rivers, creeks, ponds, and irrigation canals. Sampling was stratified to include different types of surface water sources, and site selection was based on practical accessibility, perennial flow, and relevance to agricultural water use.

To extend the temporal coverage of the analysis through January 2023, complementary data published by Chen et al. (2024) [29] were incorporated. In the second study, 1311 additional samples were analyzed from the same watersheds using a sampling design that also included a variety of surface water sources. The spatial and temporal distributions of sampling events in the Mapocho and Maipo river basins are presented in the Supplementary Materials (Figures S1 and S2).

### 2.3. Sociodemographic Data: Who Are the Affected?

To incorporate the sociodemographic dimension in the analysis of health risks associated with the presence of *Salmonella* in surface waters and to better understand who may be affected, we integrated data from sampling points that tested positive for *Salmonella* with demographic statistics projected annually by Chile’s National Institute of Statistics (INE) based on the 2017 National Population and Housing Census [28]. Only data corresponding to municipalities within the Metropolitan Region of Santiago were considered (Figure S6).

The demographic indicators used included the total number of households, average household size (calculated as the ratio between the total number of individuals living in private dwellings and the total number of households), population density (inhabitants per km^2^), total number of women and men, and age group distribution (children and youth: 0–14 years; adults: 15–64 years; older adults: 65 years and over). The definitions and calculation methods for these indicators follow the technical guidelines established by the INE [30].

Each sampling point was georeferenced at the municipal level and cross-referenced with census data for the same territorial units. This allowed for the exploration of potential associations between the presence of *Salmonella* in surface waters and the specific sociodemographic characteristics of the surrounding populations.

### 2.4. Environmental and Land Use Data Processing

To complement the microbiological and sociodemographic analyses, additional spatial data related to the environment and infrastructure were integrated to explore potential factors associated with the presence of *Salmonella* in surface water. Only data corresponding to areas surrounding the Maipo and Mapocho river basins were considered.

Two main sources of environmental information were used. First, georeferenced data on Wastewater Treatment Plants (WWTP), WWTP Discharge Points, Operational Territories, and Fire Hydrants were included, obtained from the Superintendency of Sanitary Services [31,32]. In addition to the location of the facilities, this dataset provides detailed information on discharge receptors, which represent the final destinations of the effluents. These receptors encompass a variety of natural and artificial environments, including rivers without dilution capacity, irrigation systems, dry streams, lagoons, lakes, infiltration zones, streams without dilution, natural streams, and spill sites.

Additionally, land cover and land use data were incorporated from the Catastro y Actualización de los Recursos Vegetacionales y Uso de la Tierra of the Metropolitan Region, corresponding to 2019 and mapped at a scale of 1:50,000 [33]. This dataset, developed for land-use planning and environmental monitoring purposes, classifies the territory into several categories relevant to the study area. These include forests (plantations, native, and mixed forests), Grasslands and Shrublands (shrubland-grassland mosaics, succulent shrublands, and shrublands with trees), Agricultural Lands (areas designated for agricultural use and crop-pasture rotation systems), Areas Devoid of Vegetation (rock outcrops, riverbeds, and other unvegetated zones), Urban and Industrial Areas (cities, towns, industrial zones, and mining infrastructure), Water Bodies (lakes, lagoons, reservoirs, ponds, and rivers), Permanent Snow and Glaciers, and Wetlands (vegas, bofedales, and herbaceous vegetation along watercourses).

Georeferenced data from WWTP and land use layers were cross-referenced with the sampling points that tested positive for *Salmonella*, in order to explore potential spatial relationships between microbial contamination and specific environmental features.

### 2.5. Remote Sensing-Based Environmental Variables

#### 2.5.1. Analysis of Derived Spectral Indices

A total of 12 indices were analyzed, grouped according to their relationship with key environmental variables, and calculated from the spectral bands of the Sentinel-2 satellite [34] as follows: These indices were mapped across the Maipo River Basin to assess how the surrounding conditions related to the potential presence of pathogens. We used spectral data from the Sentinel-2 SR Harmonized collection, selected for its appropriate spatial and temporal resolution [25], and all processing was performed using the Google Earth Engine (GEE) cloud-based big data platform. Only images from January 2019 to January 2023 with less than 50% cloud cover were included to preserve the spectral integrity. Preprocessing involved atmospheric correction and calculation of temporal medians for each spectral band. From these processed data, we derived indices associated with vegetation, urbanization, and water quality, which were then statistically linked to field-detected *Salmonella* presence to calibrate a predictive model using machine learning techniques.

A set of spectral indices derived from remote sensing was used as independent variables. The dependent variable was the presence (class 1) or absence (class 0) of *Salmonella*. These indices were selected because they provide complementary information on environmental characteristics that potentially influence the presence of *Salmonella* in specific geographical regions. To ensure the quality of the data, a correlation analysis was conducted on the variables, assessing the importance of each. The matrix correlation between the indices is demonstrated in Supplementary Figure S4.

##### Vegetation-Related Indices

Normalized Difference Vegetation Index (NDVI): was used to assess vegetation health by quantifying biomass and plant vigor [35]. This index allowed the mapping of vegetation density throughout the basin, with particular attention to riparian zones, which play a crucial role in contaminant filtration and the protection of water quality. High NDVI values were interpreted as areas with dense vegetation and the potential for natural pollutant mitigation [36].

SAVI (Soil Adjusted Vegetation Index) was applied as a variant of NDVI, adjusting for soil influence in areas with sparse vegetation. It was used to analyze semi-arid or disturbed regions, improve the differentiation between bare soil and vegetative cover, and contribute to a more accurate assessment of riparian ecological health [37,38].

The Enhanced Vegetation Index (EVI) was employed as an alternative to NDVI to monitor densely vegetated areas due to its reduced susceptibility to spectral saturation [39]. It is useful for detecting small variations in vegetation health in high-coverage riparian zones [40].

GCI (Green Chlorophyll Index) was used to estimate chlorophyll content in plants, serving as a direct indicator of photosynthetic activity and vegetation physiological status. This index allows the identification of deteriorated vegetation zones around sampling sites, which may affect pollutant filtration capacity or bank stability [41,42].

The Green Normalized Difference Vegetation Index (GNDVI) was applied to evaluate photosynthetic activity using the green and near-infrared bands. It was useful for detecting water stress and variations in vegetation health in both agricultural and native vegetation areas, which may influence water quality through runoff and erosion. The green normalized difference vegetation index (GNDVI) is a vegetation number that can be calculated using the green and near-infrared bands [43].

Normalized Difference Moisture Index (NDMI) was used to monitor vegetation moisture content, particularly in riparian zones near sampling points. This information is critical for understanding how moisture influences processes such as evapotranspiration and infiltration, both of which directly impact hydrological dynamics and water quality [44].

##### Water-Related Indices

Normalized Difference Water Index (NDWI): NDWI was used to detect surface water presence, delineate flood-prone areas, and monitor variations in river water levels over time [42]. It also helped identify critical regions where water quality degradation may be associated with increased contaminants and turbidity [43].

MNDWI (Modified Normalized Difference Water Index): The MNDWI is based on the normalized difference water index (NDWI), which utilizes the green and near-IR channels for water delineation. It was applied as a variant of NDWI to improve water body detection in urban environments [44]. It is particularly useful for mapping water bodies in areas where the traditional NDWI may fail due to interference from artificial surfaces, such as concrete and asphalt [45].

The Automated Water Extraction Index (AWEI) was used to improve water detection in areas with mixed surfaces, such as shadows and vegetation, which often interfere with traditional water indices [46]. This index is especially useful in zones affected by extreme weather events or marked seasonal changes.

The LSWI (Land Surface Water Index) was employed to identify the surface moisture content in soil and vegetation [45]. Its application enabled the identification of areas with high surface moisture associated with runoff, standing water accumulation, or potential hotspots for pathogen proliferation, such as *Salmonella*.

##### Urban Development-Related Indices

The Normalized Difference Built-up Index (NDBI) was used to identify urbanized areas and human expansion within the basin. This index was key in assessing whether sampling points were influenced by urbanization, considering its potential to increase pollutant loads and surface impermeability factors that enhance runoff and the contamination of water bodies. The Composite Study Area had a significant Urban-Built-Up area, but no efforts were made to monitor and provide the composite area artificially [47,48,49,50].

NDTI (Normalized Difference Tillage Index) was applied to detect soil preparation practices and agricultural cover [51]. In this study, NDTI was used to identify agricultural zones where fertilizer application or soil erosion may act as contamination sources that affect water quality.

### 2.6. Detection of Salmonella Based Index Derived Remote Sensing

Statistical tests and calibration procedures were conducted to develop a predictive model based on satellite images to identify the environmental conditions associated with the presence of *Salmonella* in surface waters. The analysis used 12 spectral indices as independent variables and the presence (class 1) or absence (class 0) of *Salmonella*, determined from field samples, as the dependent variable. Temporal variation was not considered in this stage. Although the indices do not directly detect pathogens, their combined use offers a robust characterization of environmental conditions that can help explain and predict *Salmonella* occurrences, providing useful input for surveillance and risk mitigation strategies.

To prepare the data, all variables were standardized to ensure comparability across scales. The dataset was then randomly split, with 80% used for training and 20% for testing. We implemented a two-stage evaluation procedure to robustly assess the model’s performance. First, the hold-out test set (20% of the data) was kept completely separate and used only once at the end to estimate the out-of-sample performance. Second, within the remaining 80% training set, we applied stratified 5-fold cross-validation: the training set was partitioned into five folds, and in each iteration, the model was trained on four folds (≈64% of the total data) and validated on the fifth (≈16%). We repeated this process five times so that each observation served exactly once as validation, and then averaged the metrics (accuracy, recall, F1-score, and AUC) across the folds to guide model selection and hyperparameter tuning.

Due to class imbalance (1147 records of *Salmonella* absence versus 704 of presence), we applied the SMOTETomek technique, which combines SMOTE (Synthetic Minority Oversampling Technique) to generate synthetic examples of the minority class, with Tomek links to remove ambiguous examples from the majority class. For model selection, we used the LazyPredict library to perform an initial screening of multiple classification algorithms without prior hyperparameter tuning. This process identified the ExtraTreesClassifier (Table 1) as the model with the best preliminary performance. ExtraTrees is an ensemble learning method that constructs multiple decision trees using random subsets of both training data and predictor variables.

### 2.7. Salmonella Prevalence

Considering the *Salmonella* sampling points, the prevalence of *Salmonella* presence within the study area was analyzed, with the aim of identifying which sites, during the collection period, are potential sites with the presence of the pathogen. Considering the spatial distribution of the samples, spatial interpolation methods were applied to estimate the areas that were most at risk of contamination. For modeling, we used a consolidated database containing information on the presence (1) or absence (0) of bacteria at each site.

To analyze the spatial distribution of *Salmonella* prevalence within the study area, we used the Inverse Distance Weighting (IDW) interpolation method, a robust technique for estimating values at unsampled locations based on the influence of surrounding sample points [52]. The IDW method assumes that the weight of each sample point is inversely correlated with its distance from the prediction location, making it well-suited for environmental studies in which the distribution of sample points is irregular [53].

To apply the interpolator, the power parameter (p) was considered, which was determined through sensitivity tests to ensure an adequate balance between smoothing and detailing the interpolation, resulting in a value of 2.5. Interpolation was performed for data combined over time and separately by year, allowing the identification of persistent patterns and temporal variations in contamination.

**Figure 2:**
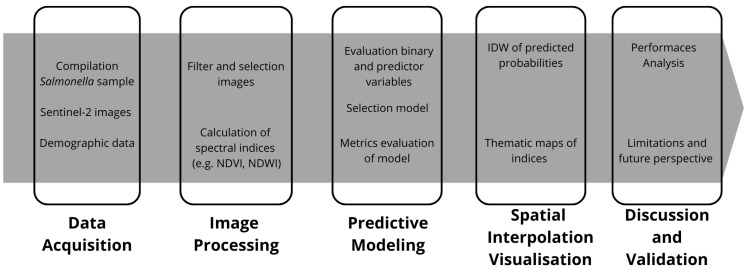
Workflow summary diagram of the methodological framework.

As a summary of the workflow, Figure 2 presents a diagram that illustrates each phase of the methodology, from image acquisition and preprocessing to data integration and interpretation.

## 3. Results and Discussion

### 3.1. Demographic Analysis and Its Association with the Presence of Salmonella

**Figure 3:**
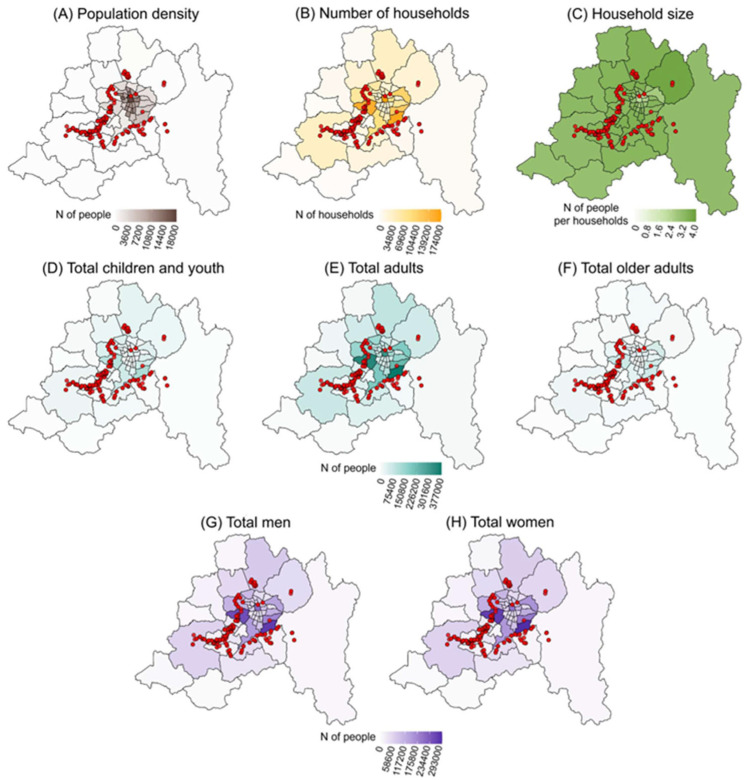
Spatial distribution of sociodemographic indicators across municipalities in the Metropolitan Region of Santiago, Chile. Each map represents a different variable measured at the municipal level: (**A**) population density, (**B**) total number of households, (**C**) average household size, population by age group ((**D**) 0–14 years, (**E**) 15–64 years, and (**F**) 65 years or older), (**G**) total number of men, and (**H**) total number of women. The intensity of the color gradient reflects the relative value of each indicator for each municipality. Red dots indicate the locations where *Salmonella-positive* water samples were detected.

Based on an exploratory analysis that integrated microbiological data on the presence of *Salmonella* in surface waters with sociodemographic indicators, no clear correlations were identified between the variables considered. Specifically, no consistent patterns were observed linking positive sampling points with demographic characteristics such as average family size, population density, gender distribution, or age structure (Figure 3). Although some municipalities with higher population densities reported isolated detections of *Salmonella*, no statistical relationship was found with other sociodemographic indicators.

Previous studies have documented a significant association between population growth and water quality deterioration [54]. These studies suggest that when critical density thresholds, such as 2375 inhabitants per km^2^, are exceeded, there is a sharp increase in parameters such as biochemical oxygen demand (BOD) and total coliform concentrations, indicating a decline in water quality [54]. These conditions can potentially occur near human settlements, particularly in informal communities. In Chile, these informal settlements, known as *campamentos*, are defined as precarious communities of eight or more households that occupy land irregularly and lack at least one basic service, usually sewage systems. Many *campamentos* are located near river basins, often on marginal or flood-prone lands (Figure S7). In recent years, their numbers have grown rapidly due to rising housing costs, a deepening housing deficit, and broader social and economic crises [55,56,57,58]. By 2023, approximately 114,000 households, or about 2% of the national population, were living in *campamentos*, a sharp increase from 47,000 in 2019. Environmental and infrastructural factors may play a role in the distribution of *Salmonella* in aquatic environments [6,59,60].

### 3.2. Assessing the Role of Erosion and WWTP Proximity in Salmonella Detection

**Figure 4:**
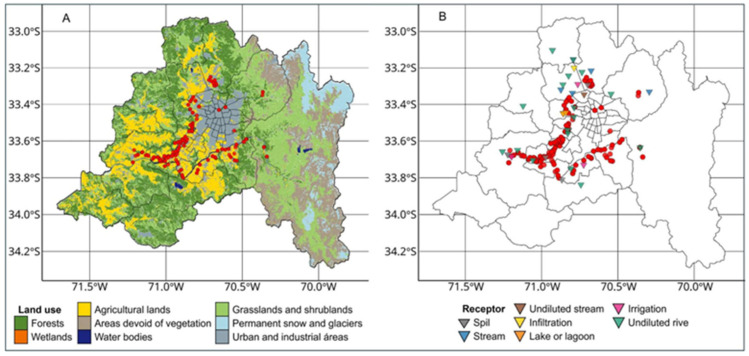
Environmental variables in relation to *Salmonella* presence. This figure shows the spatial distribution of *Salmonella*-positive sampling points (in red) over two environmental layers. Panel (**A**) shows *Salmonella*-positive sites (in red) overlaid on the regional land use classification. Panel (**B**) shows the locations of *Salmonella*-positive points (red) in relation to wastewater treatment plants (inverted triangles) categorized by type.

The spatial analysis of *Salmonella*-positive sampling points in relation to land use and wastewater treatment infrastructure revealed certain patterns of interest, although these were not conclusive (Figure 4). Although several detections occurred in urban, agricultural, and wastewater treatment plant (WWTP) discharge areas, such spatial coincidences were neither absolute nor uniformly distributed across the basin.

A number of positive sampling sites were located within urban and industrial land-use zones and near agricultural areas. This distribution may be related to intensive land use practices that promote surface runoff, the use of contaminated water for irrigation, or the accumulation of fecal waste, all of which have previously been associated with *Salmonella* dissemination in aquatic environments [61,62]. However, similar environmental settings were also observed without any positive detection, suggesting that land use alone is not a definitive predictor of contamination.

More notably, there was spatial clustering of positive sites near WWTP or their discharge areas. This pattern aligns with the findings of Levantesi et al. (2012) [63], who reported that *Salmonella* can persist even after advanced secondary wastewater treatment, indicating that insufficiently treated effluents may contribute to contamination in surface waters. Similarly, Liu et al. (2018) [60] highlighted that water used in agriculture, especially when drawn from surface sources near urban zones or WWTP, can serve as a transmission route for *Salmonella* through irrigation and other agricultural practices.

Despite these spatial associations, a direct causal relationship between proximity to WWTP, land use types, and *Salmonella* presence cannot be firmly established, as positive samples were also recorded in areas lacking such features. Thus, the results suggest a geospatial trend that may be shaped by a combination of factors, including the efficiency of wastewater treatment, seasonal variations, local hydrological conditions, and wildlife interactions. Further studies with higher spatial and temporal resolutions are needed to deepen our understanding of these potential relationships.

Overall, these findings reinforce the notion that the presence of *Salmonella* in surface waters cannot be fully explained by isolated, sociodemographic, or geographic variables. Instead, an integrated analysis that considers landscape configuration, point-source pollution, and local agricultural practices is essential to better understand the dynamics of microbial contamination in urban and peri-urban environments.

### 3.3. Remote Sensing

**Figure 5:**
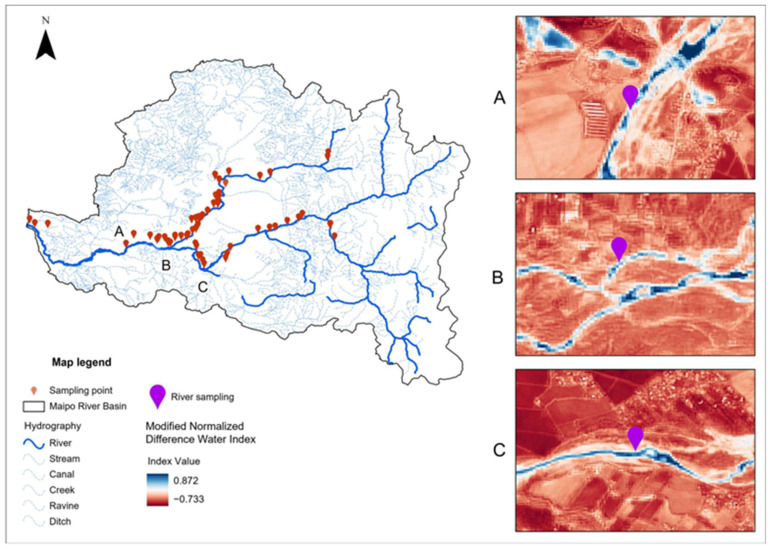
Location of water sampling points within the Maipo River hydrographic basin and examples of NDWI images for points 1, 2, and 3 in November 2019.

Analysis of the spectral indices derived from Sentinel-2 images revealed environmental patterns associated with the presence of *Salmonella*. While all samples were collected from aquatic environments (Figure 5), the spatial resolution of the satellite imagery (10 m) means that the analyses captured not only water but also the characteristics of the surrounding areas [64]. These environmental features, reflected in the spectral data, can be inferred and statistically linked to the presence of pathogens.

**Figure 6:**
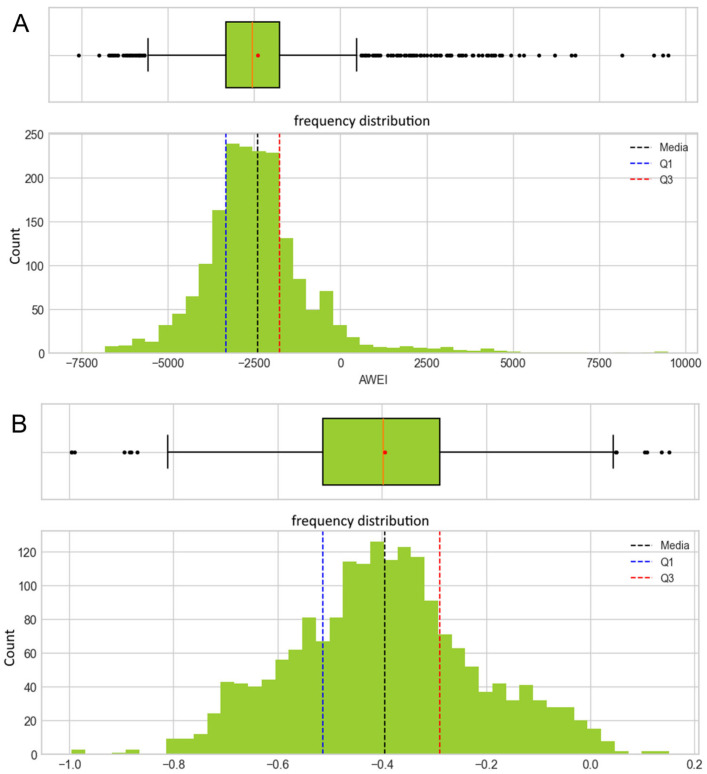
Distribution of Automated Water Extraction Index (AWEI) (**A**) and Normalized Difference Water Index (NDWI) (**B**). The graphs show the box plots and histograms of the distribution of the collected index values. The blue dotted line represents the first quartile, the red dotted line represents the third quartile, and the black dotted line represents the mean.

A total of 1851 water samples were collected between 2019 and 2023, of which 1147 were negative and 704 were positive for *Salmonella* (Figure S3). The spectral index analysis showed that NDWI and AWEI (Figure 6), both linked to the presence of water, had the largest correlation with *Salmonella*-positive samples, highlighting areas of greater contamination risk. The descriptive statistics and summary of the other spectral indices studied are presented in the Supplementary Materials (Figures S4 and S5). These areas are characterized by significant variations in humidity and water flow. During winter (June–August), rainfall over the steep Andean catchments drives rapid increases in river flow, diluting microbial concentrations, even as sediment mobilization raises turbidity and surface moisture signals captured by NDWI and AWEI. In contrast, late spring and early summer flows are sustained by snowmelt, keeping water temperatures low and spectral water indices high, but by mid-summer (December–February), both precipitation and snowmelt wane, leading to lower, warmer flows that favor *Salmonella* persistence while diminishing NDWI and AWEI values (see time series of temperature and precipitation in the Supplementary Materials).

The result of balancing was a balanced dataset with 807 samples for each class. The samples for testing and training were separated from this set. The results of applying the ExtraTreesClassifier model, as shown in Table 2, show an overall accuracy of 59.2 %.

**Figure 7:**
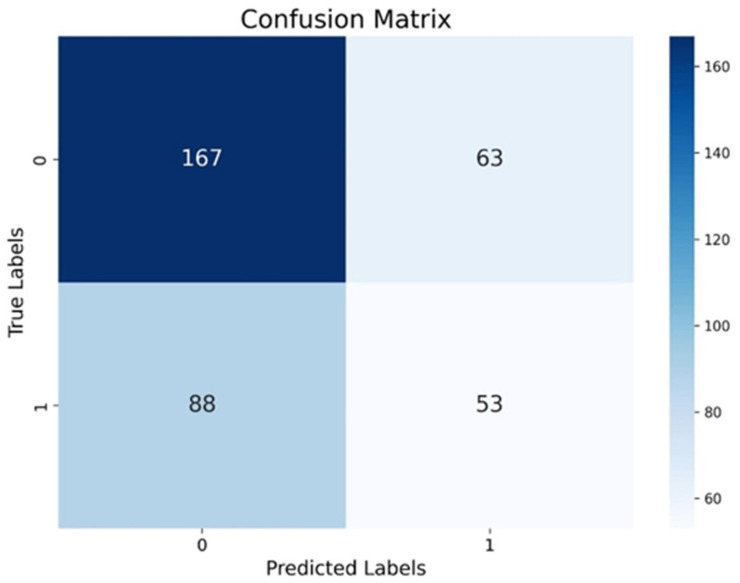
Confusion matrix. The graph summarizes the results of the comparison between the model’s predictions and the actual values.

The results suggest that the model performs better in classifying class 0 (absence of *Salmonella*), with higher precision, recall, and F1-score values. The model’s confusion matrix (Figure 7) reflects the model’s greater ease in recognizing negative samples, in contrast to its lower sensitivity in detecting positive cases (class 1), which is a relevant limitation when the objective is to conduct epidemiological surveillance.

It is important to emphasize that the aim of our study was to establish an effective prediction structure rather than to conduct an in-depth exploratory analysis of the variables. Accordingly, we prioritized methodological efficiency and prediction performance. To achieve this, we adopted a robust and efficient initial model selection strategy using the LazyPredict library, which allowed a comprehensive and automated comparison of various classification algorithms without the need for prior adjustment of hyperparameters. Among the tested models, the ExtraTreesClassifier exhibited the best performance. This ensemble method builds multiple random decision trees, which then perform an implicit sensitivity analysis to evaluate how each resource contributes to reducing impurity during training.

### 3.4. Spatial Analysis of Salmonella Prevalence

**Figure 8:**
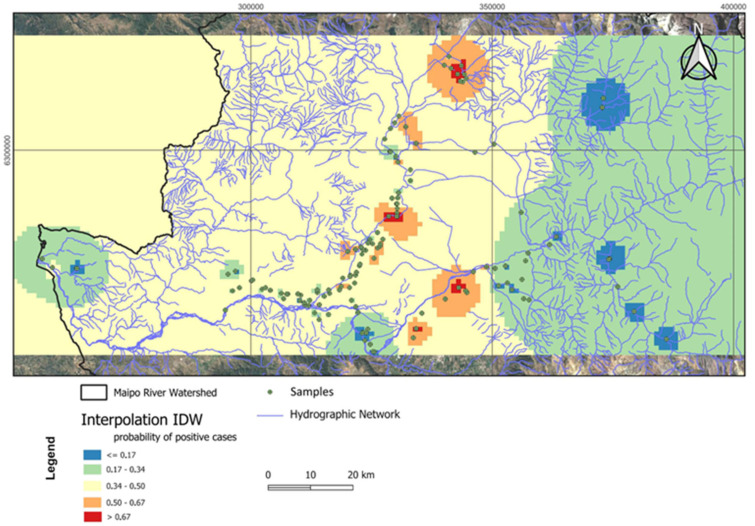
Interpolated surface showing the spatial probability of *Salmonella* occurrence across the study area using the Inverse Distance Weighting (IDW) method. The map highlights zones of higher and lower risk within the watershed, revealing the spatial patterns of potential contamination.

We analyzed the spatial distribution of *Salmonella* prevalence using IDW. The IDW-generated surface allowed us to identify areas with the highest probability of *Salmonella* occurrence, highlighting regions of greater risk within the watershed. With input values coded as ‘1’ for presence and ‘0’ for absence, the interpolated regions with values approaching 1 indicated a higher likelihood of *Salmonella* presence, while values closer to 0 corresponded to areas with lower contamination risk (Figure 8).

The points with the highest prevalence or hotspots were associated with different land-use characteristics. The northern hotspot is located in a residential area with single-family homes and higher income levels than the other hotspots. Point 2, which is more centrally located, is situated near an effluent treatment plant that uses an activated sludge system. The third hotspot, located further south, is in an agricultural zone. These findings suggest that the presence of *Salmonella* in the study area is likely influenced by surrounding environmental factors, such as land use and infrastructure.

Despite its potential in terms of results, it should be noted that IDW has its own model limitations due to its sensitivity to outliers and dependence on the distribution of the sample [65,66], which we have partially solved by limiting the search radius and adjusting the parameter settings.

## 4. Conclusions

*Salmonella* causes millions of cases annually. Efficient and timely water quality surveillance is therefore critical. However, routine monitoring for pathogens like *Salmonella* is time-consuming, requires specialized equipment, and faces significant limitations, including coverage gaps, delayed data, and limited accessibility.

Given the growing impact of climate change and the increasing frequency of extreme weather events, the integration of satellite data with traditional monitoring methods is becoming critical. The implementation of early warning systems based on remote sensing can enhance decision-making in public health and water resource management, promoting more effective and sustainable water quality protection strategies.

In this study, we explored the potential of remote sensing-derived spectral indices for detecting environmental conditions associated with the presence of pathogens in water samples. Although the overall accuracy of 59% is considered moderate, a detailed analysis by class reveals an asymmetry in performance that should be considered in the practical application of the model. We assessed the spatial distribution of *Salmonella* within the watershed to identify regions with a heightened risk of contamination and their relationship with the surrounding environmental conditions. We found that *Salmonella* prevalence in surface waters is primarily influenced by environmental factors, particularly proximity to wastewater treatment plants, rather than by sociodemographic characteristics. Remote sensing and spatial analysis techniques, such as spectral indices and IDW interpolation, effectively identified areas with higher contamination risk, highlighting the potential of these methods for monitoring and managing water quality.

This study had some limitations, mainly related to the *Salmonella* data sample. The lack of a more extensive temporal sequence and infrequent collections over the years hindered the analysis of the seasonal patterns. Additionally, low river flow and extreme drought during the sampling period further limited data representativeness and the effectiveness of satellite imagery.

Several studies have demonstrated the promising potential of remote sensing for applications in lakes and other water bodies, which have already been successfully applied by other authors for monitoring water quality and biological indicators. Although the methodologies are promising for the development of a surveillance system for water-related diseases, the main contribution of the research may lie more in revealing difficulties and indicating obstacles that need to be overcome in the future rather than in presenting an immediately available monitoring tool. For example, creating a comprehensive field database to calibrate remote sensing results is extremely important. Such a database would provide essential inputs for better quality control, increasing the accuracy and effectiveness of monitoring tools for public health intervention.

This research highlights the potential of spectral indices as environmental predictors for *Salmonella* risk mapping and the challenges and difficulties that must be overcome for remote sensing to represent a meaningful contribution to environmental and epidemiological surveillance systems.

## Figures and Tables

**Table 1:** Flowchart methodology using ExtraTreesClassifier.

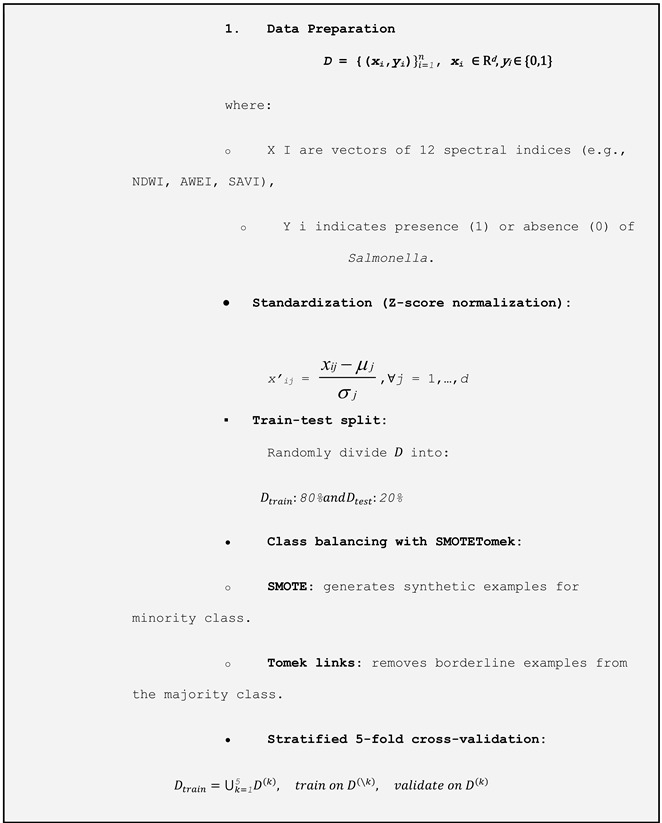 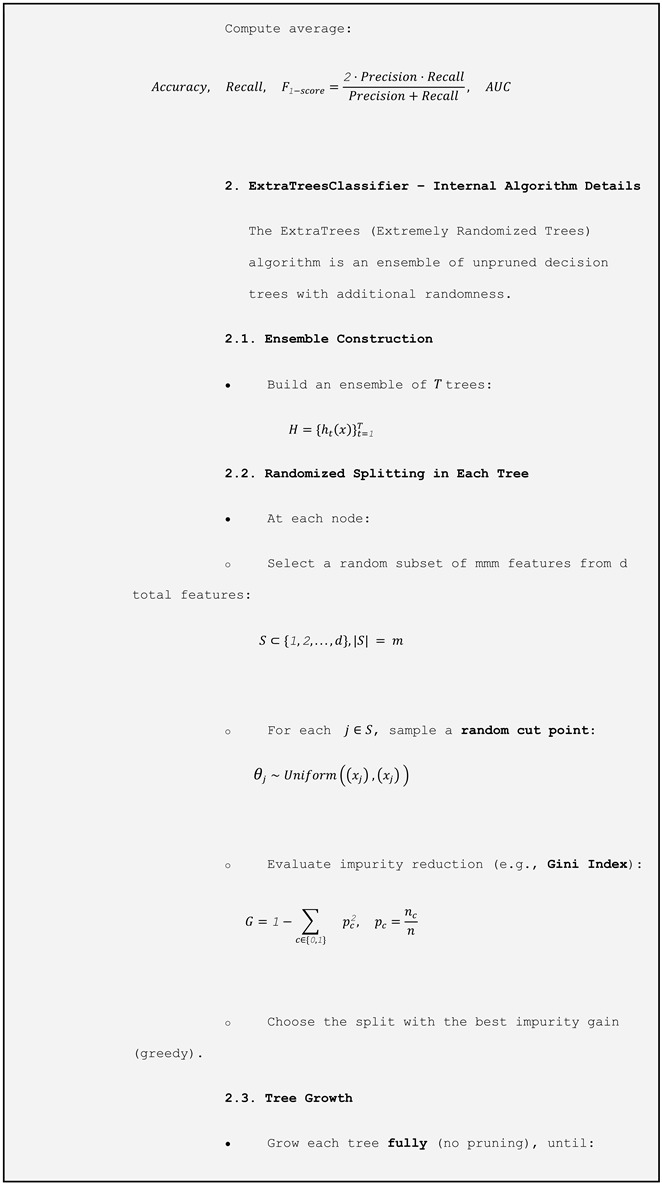 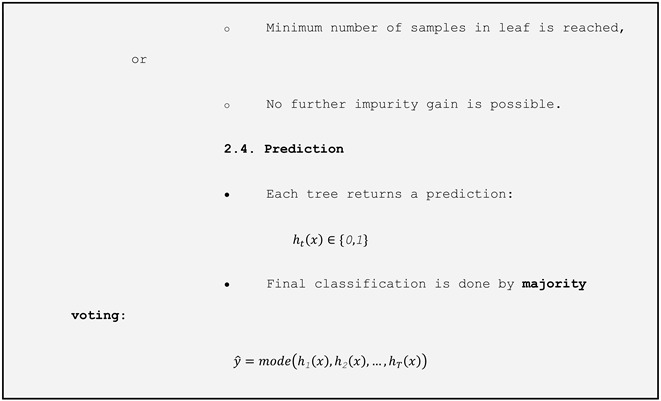

**Table 2:** Output metrics of the ExtraTreesClassifier model.

Presence *Salmonella*	Precision	Recall	f1-Score	Support
**0**	0.65	0.73	0.69	230
**1**	0.46	0.38	0.41	141
**Accuracy**			0.59	371

## Data Availability

The original contributions presented in this study are included in the article/Supplementary Material. Further inquiries can be directed to the corresponding author.

## Supplementary Materials

The following supporting information can be downloaded at: https://www.mdpi.com/article/10.3390/microorganisms13071539/s1. Figure S1. Sampling of the Mapocho River during the evaluation years. Figure S2. Sampling of the Maipo River during the evaluated years. Figure S3. Occurrence of *Salmonella* in water samples. The dataset used contained 1147 records classified as *Salmonella* absence and 704 as presence. Figure S4. Data preprocessing and balancing. Figure S5: Histogram distribution of the different spectral indices. Figure S6. Communes of the Metropolitana region of Santiago, Chile. Figure S7. Informal settlements are located near the study area. Figure S8. Temporal series of precipitation (A) and mean temperature (B) at a point (−70.58E; −33.62N) in the surroundings of the Maipo River. Table S1. Descriptive statistics for the spectral indices studied. Table S2. Equations for Index.
