# Supplementary material for: Challenges and Potential of Remote Sensing for Assessing Salmonella Risk in Water Sources: Evidence from Chile
Source: Microorganisms. 2025 Jun 30;13(7):1539. doi: 10.3390/microorganisms13071539 (PMC12299355; doi:10.3390/microorganisms13071539)
Supplement: Supplementary file 1 [file microorganisms-13-01539-s001.zip › microorganisms-3623289-supplementary.pdf]

Supplementary Materials

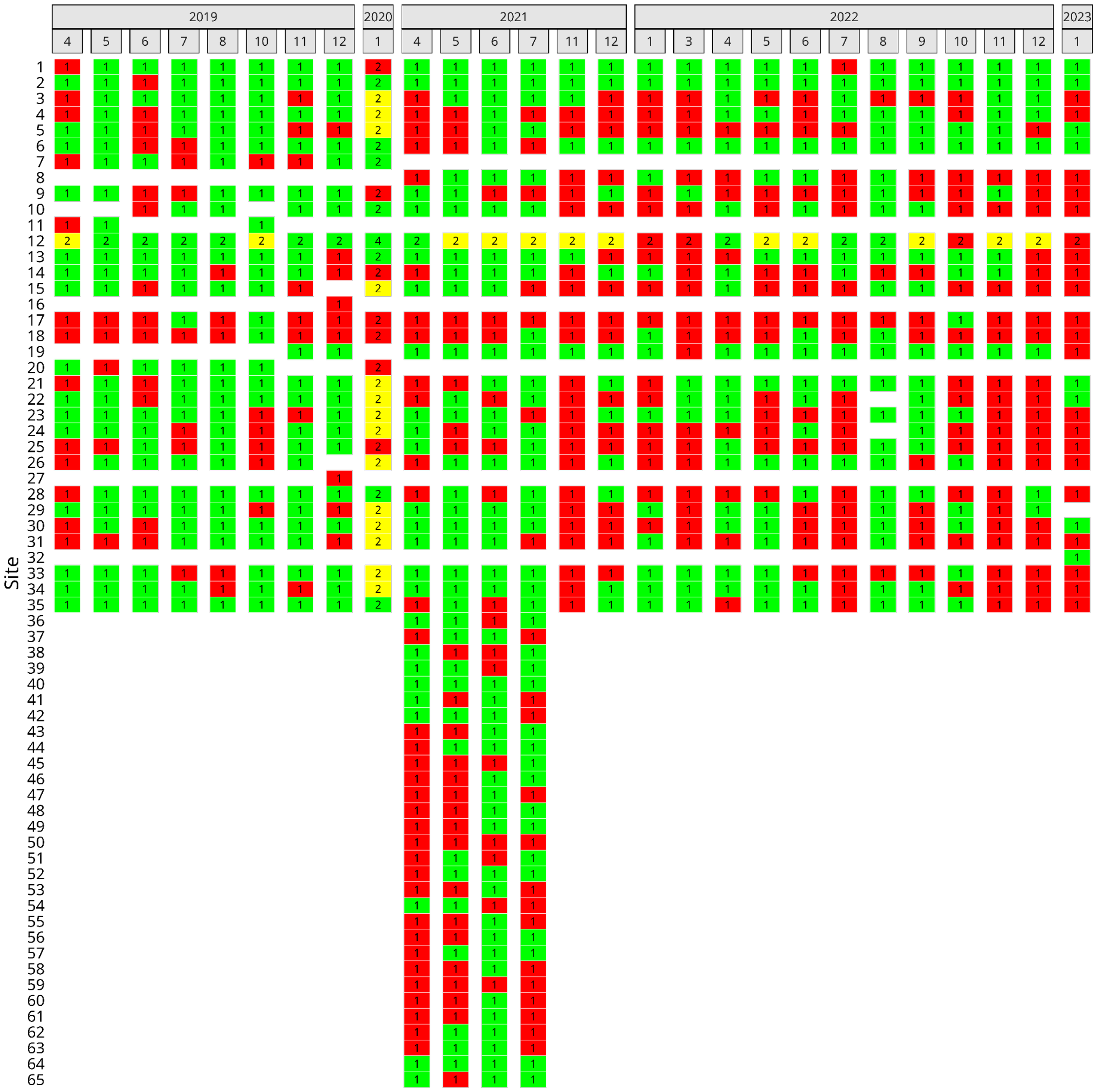

Figure S1. Sampling of the Mapocho River during the evaluated years, with each year subdivided by month. Sampling sites were defined based on geographic proximity: points located within 100 meters of each other were grouped as a single site, while those beyond this distance were considered separate sites. The color of each tile indicates whether *Salmonella* was detected at the site: red indicates presence, green indicates absence, and yellow indicates detection in some seasons but not others. The number inside each tile represents the total number of samples collected at that site during the indicated month. White tiles indicate that no samples were collected at that site and time due to differences in river flow, site inaccessibility, or other logistical limitations.

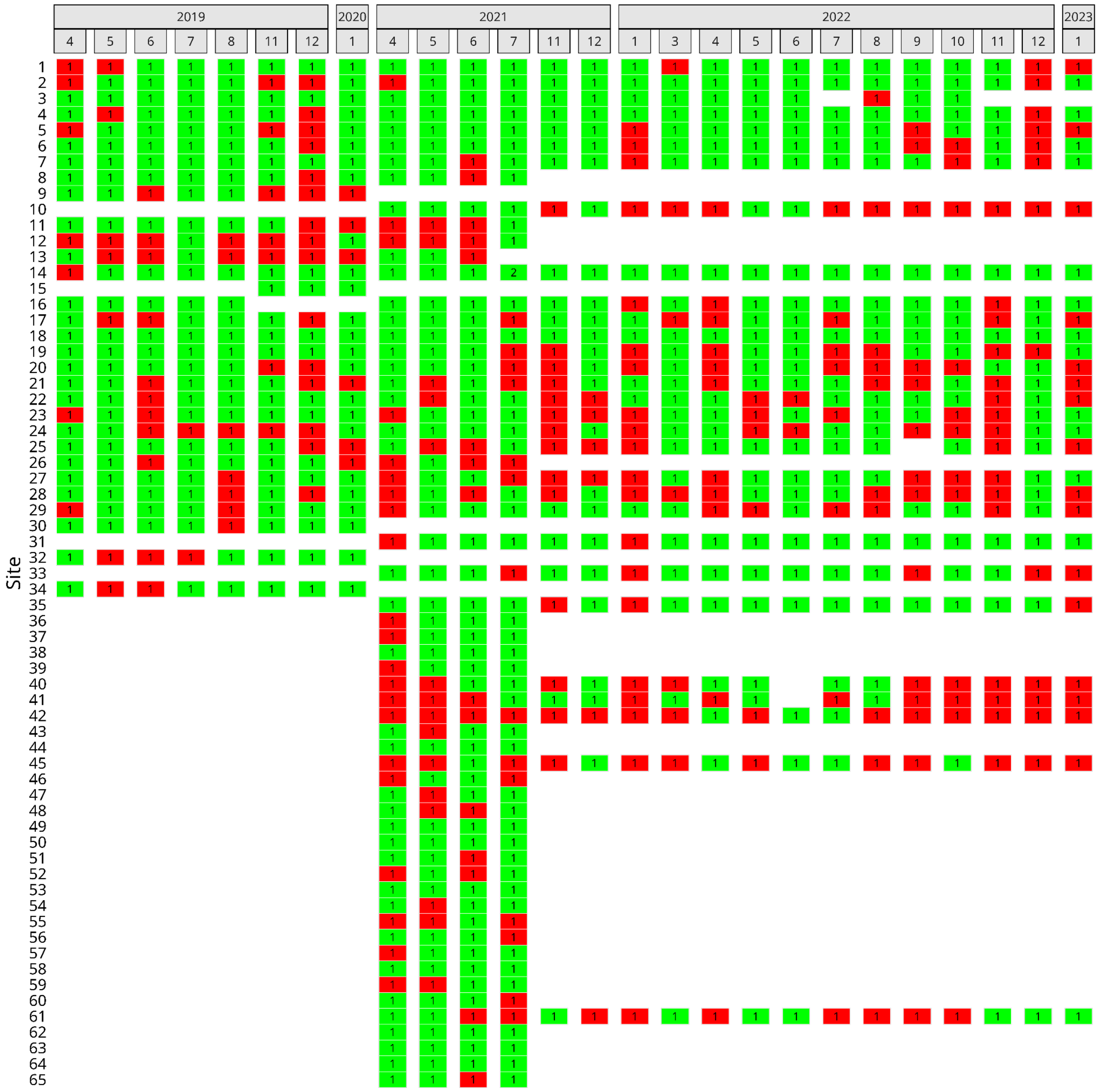

Figure S2. Sampling of the Maipo River during the evaluated years, with each year subdivided by month. Sampling sites were defined based on geographic proximity: points located within 100 meters of each other were grouped as a single site, while those beyond this distance were considered separate sites. The color of each tile indicates whether *Salmonella* was detected at the site: red indicates presence and green absence. The number inside each tile represents the total number of samples collected at that site during the indicated month. White tiles indicate that no samples were collected at that site and time due to differences in river flow, site inaccessibility, or other logistical limitations.

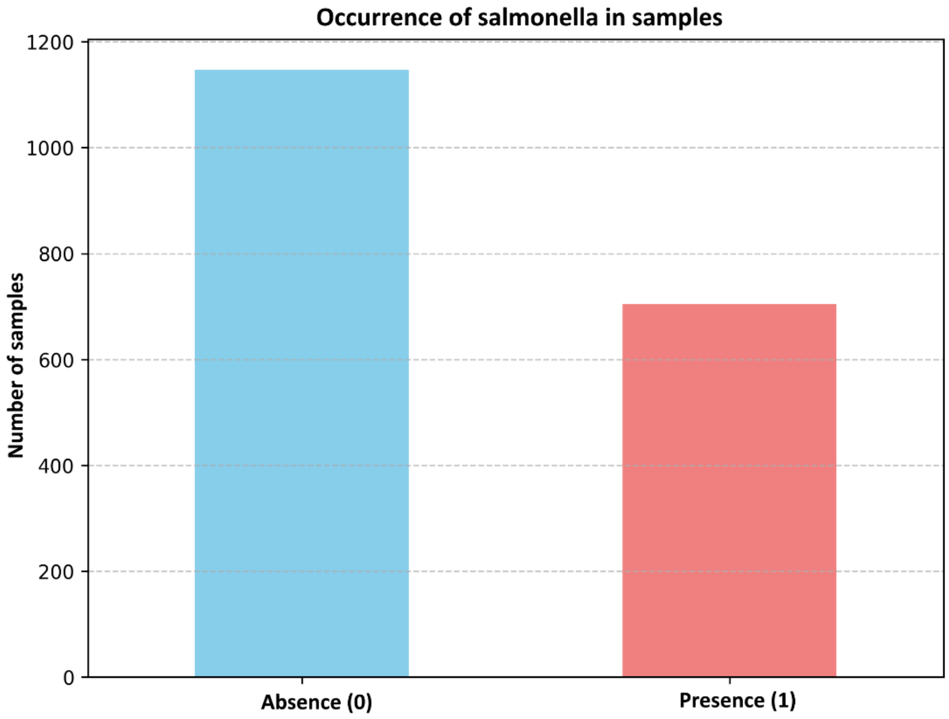

Figure S3 – Salmonella ocurrence in water sample. The dataset used contained 1,147 records classified as Salmonella absence and 704 as presence

Data pre-processing and balancing

A set of spectral indices derived from remote sensing were used as independent variables: AWEI, EVI, GCI, GNDVI, LSWI, MNDWI, NDBI, NDTI, NDWI and SAVI. The dependent variable was the presence (class 1) or absence (class 0) of Salmonella.

These indices were selected because they provide complementary information on environmental characteristics that potentially influence the presence of Salmonella in specific geographical areas.

To ensure the quality of the data found, a correlation analysis was carried out on the variables, assessing the importance of each one (Figure S4).

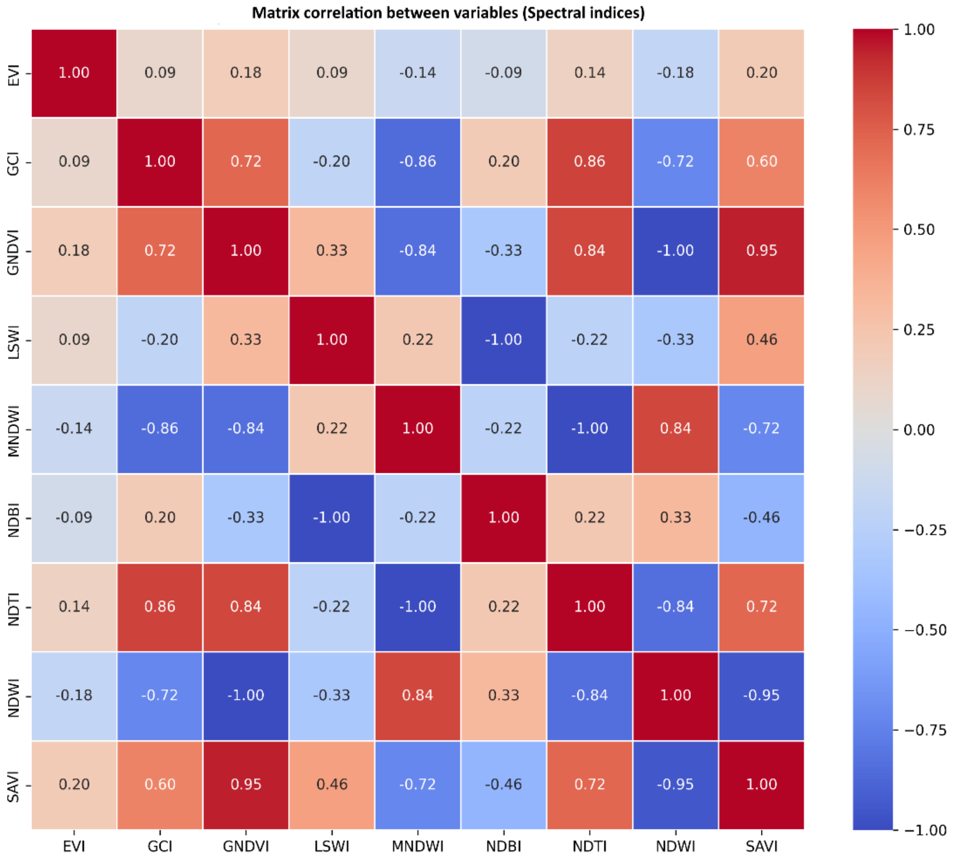

Figure S4: Data pre-processing and balancing

Descriptive statistics and summary for the spectral indices studied

In the process of cleaning the data, no missing values were identified, nor were any values considered outliers removed, since there was not enough statistical evidence to justify their exclusion. The following graphs show the histogram distribution of the different spectral indices used.

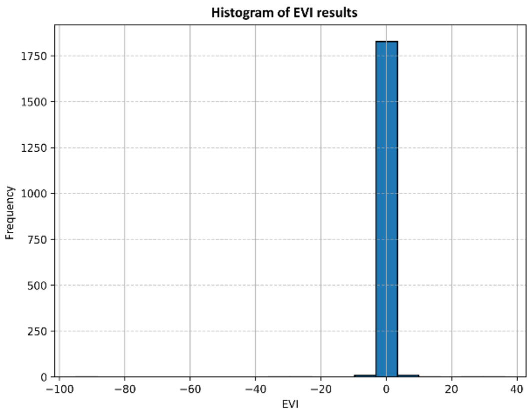
Histogram of EVI results
Frequency
EVI

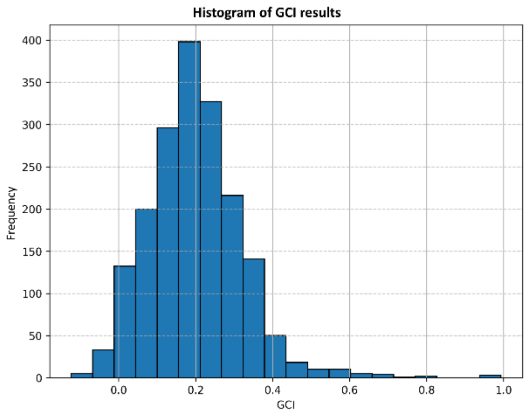
Histogram of GCI results
Frequency
GCI

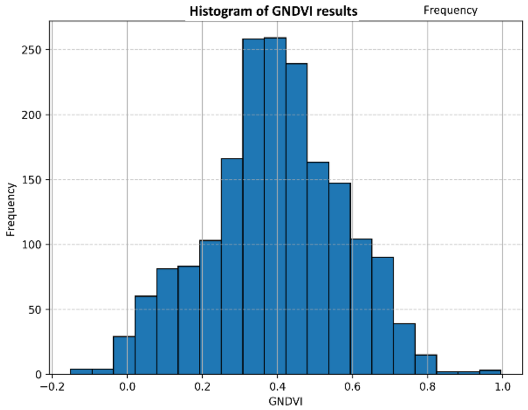
Histogram of GNDVI results
Frequency
Frequency
GNDVI

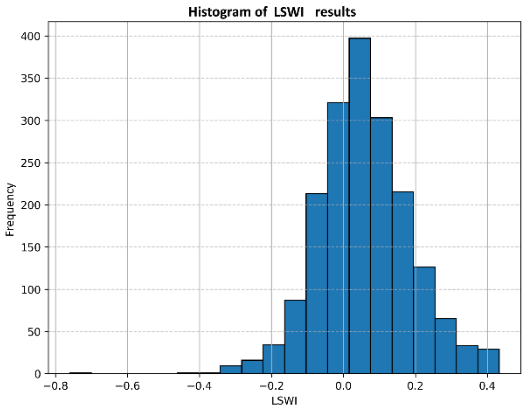
Histogram of LSWI results
Frequency
LSWI

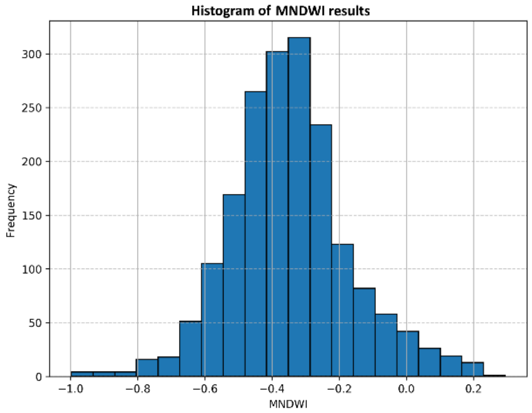
Histogram of MNDWI results
Frequency
MNDWI

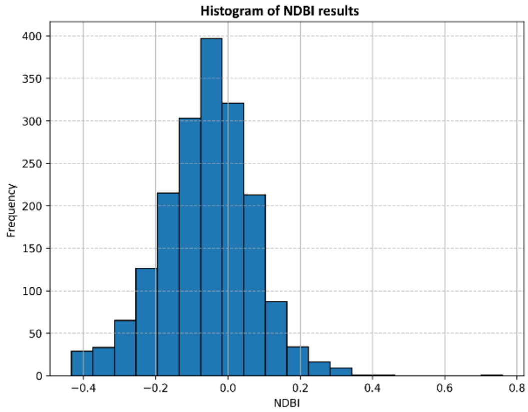
Histogram of NDBI results
Frequency
NDBI

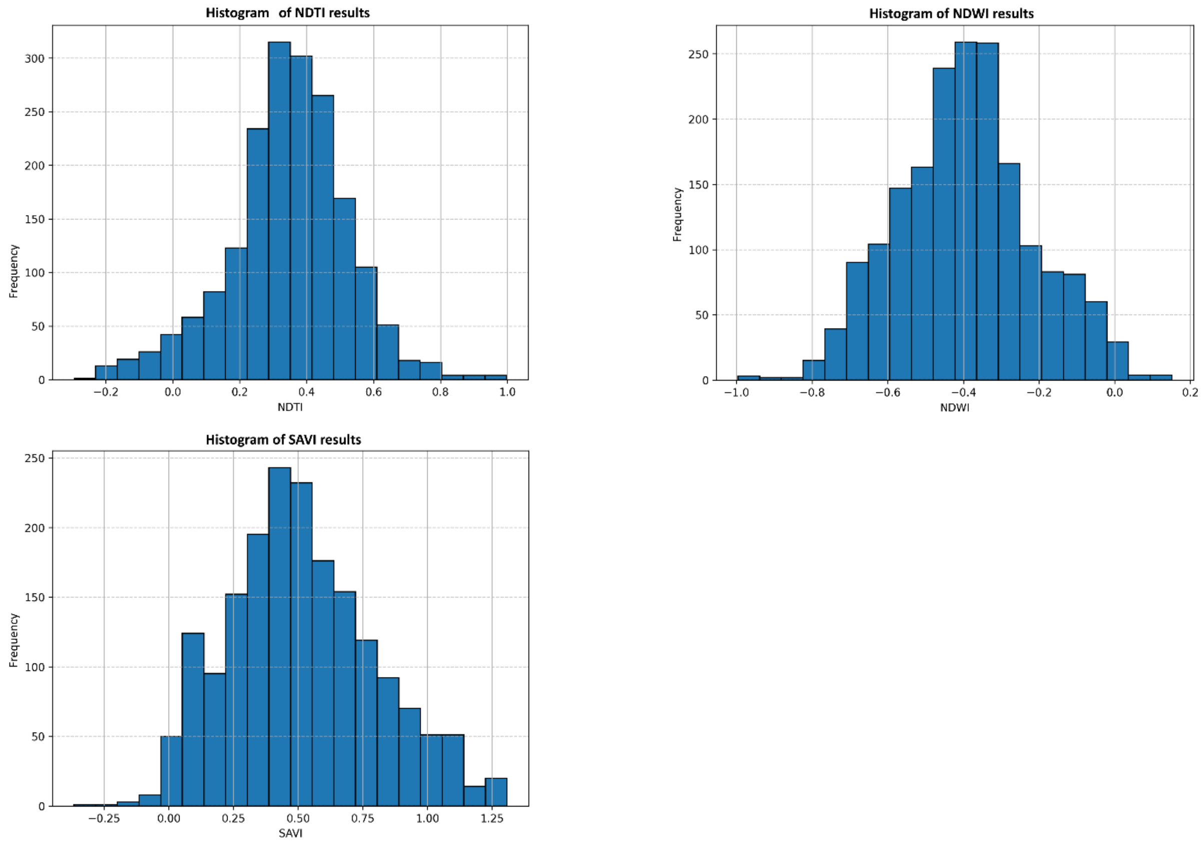

Figure S5: Histogram distribution of the different spectral indices used.

Table S1: Descriptive statistics for the spectral indices studied

		Absence	Presence
NDWI	count	1147	704.000
	mean	-0.389	-0.402
	std	0.182	0.176
	min	-0.996	-0.881
	25%	-0.506	-0.522
	50%	-0.385	-0.409
	75%	-0.284	-0.299
	max	0.137	0.151
SAVI	count	1147	704
	mean	0.506	0.525
	std	0.285	0.286
	min	-0.212	-0.366
	25%	0.313	0.336
	50%	0.481	0.499
	75%	0.682	0.714
	max	1.308	1.288
EVI	count	1147	704
	mean	0.737	0.884
	std	3.156	2.057
	min	-94.923	-30.532
	25%	0.504	0.518
	50%	0.775	0.825
	75%	1.188	1.195
	max	23.682	36.201
GCI	count	1147	704
	mean	0.197	0.203
	std	0.127	0.114
	min	-0.123	-0.074
	25%	0.110	0.138
	50%	0.191	0.196

	75%	0.267	0.267
	max	0.993	0.792

		Absence	Presence
GNDVI	count	**1147**	704
	mean	0.389	0.402
	std	0.182	0.176
	min	-0.137	-0.151
	25%	0.284	0.299
	50%	0.385	0.409
	75%	0.506	0.522
	max	0.996	0.881
LSWI	count	1147	704
	mean	0.053	0.067
	std	0.131	0.123
	min	-0.760	-0.317
	25%	-0.029	-0.015
	50%	0.047	0.060
	75%	0.132	0.147
	max	0.433	0.434
MNDWI	count	1147	704
	mean	-0.344	-0.348
	std	0.184	0.168
	min	-0.998	-0.926
	25%	-0.458	-0.451
	50%	-0.354	-0.352
	75%	-0.247	-0.264
	max	0.295	0.227
NDBI	count	1147	704
	mean	-0.053	-0.067
	std	0.131	0.123
	min	-0.433	-0.434
	25%	-0.132	-0.147

	**50%**	-0.047	-0.060
	**75%**	0.029	0.015
	**max**	0.760	0.317
**NDTI**	**count**	1147	704
	**mean**	0.344	0.348
	**std**	0.184	0.168
	**min**	-0.295	-0.227
	**25%**	0.247	0.264
	**50%**	0.354	0.352
	**75%**	0.458	0.451
	**max**	0.998	0.926

To ensure homogeneity of scale between the predictor variables, the data was standardized using the StandardScaler technique, which transforms the variables into a distribution with zero mean and unit standard deviation. Subsequently, the set was randomly partitioned into 80% for training and 20% for testing.

Due to the imbalance between the classes, the SMOTETomek technique was applied, which combines oversampling of the minority class using SMOTE (Synthetic Minority Over-sampling Technique) with undersampling of the majority class using Tomek links. The result was a balanced set with 807 samples for each class.

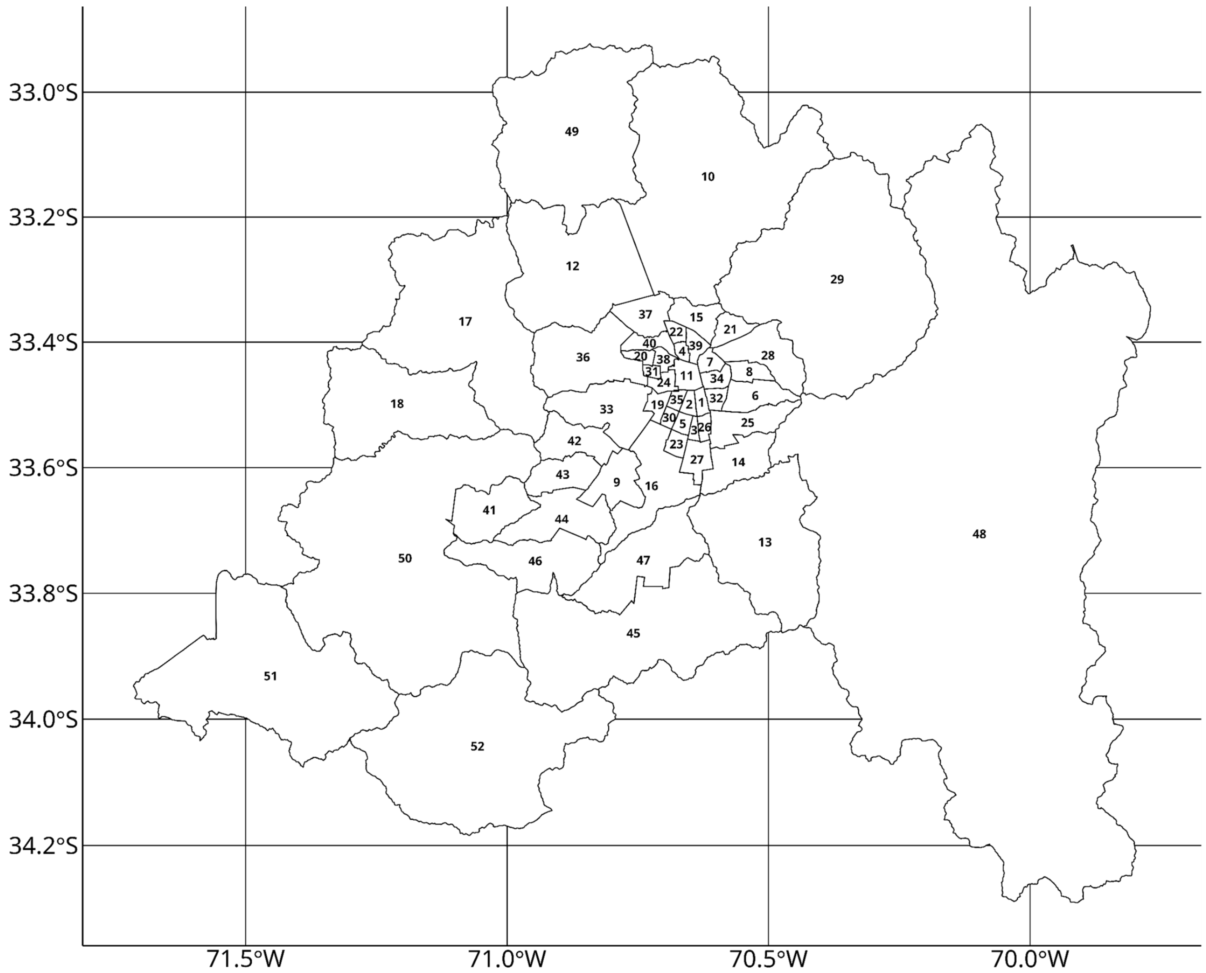

1	San Joaquín	14	Puente Alto	27	La Pintana	40	Renca
2	San Miguel	15	Huechuraba	28	Las Condes	41	El Monte
3	San Ramón	16	San Bernardo	29	Lo Barnechea	42	Padre Hurtado
4	Independencia	17	Curacaví	30	Lo Espejo	43	Peñaflor
5	La Cisterna	18	María Pinto	31	Lo Prado	44	Talagante
6	Peñalolén	19	Cerrillos	32	Macul	45	Paine
7	Providencia	20	Cerro Navia	33	Maipú	46	Isla de Maipo
8	La Reina	21	Vitacura	34	Ñuñoa	47	Buin
9	Calera de Tango	22	Conchalí	35	Pedro Aguirre Cerda	48	San Jose de Maipo
10	Colina	23	El Bosque	36	Pudahuel	49	Tiltil
11	Santiago	24	Estación Central	37	Quilicura	50	Melipilla
12	Lampa	25	La Florida	38	Quinta Normal	51	San Pedro
13	Pirque	26	La Granja	39	Recoleta	52	Alhué

Figure S6. Communes of the Metropolitana region of Santiago of Chile

A B C

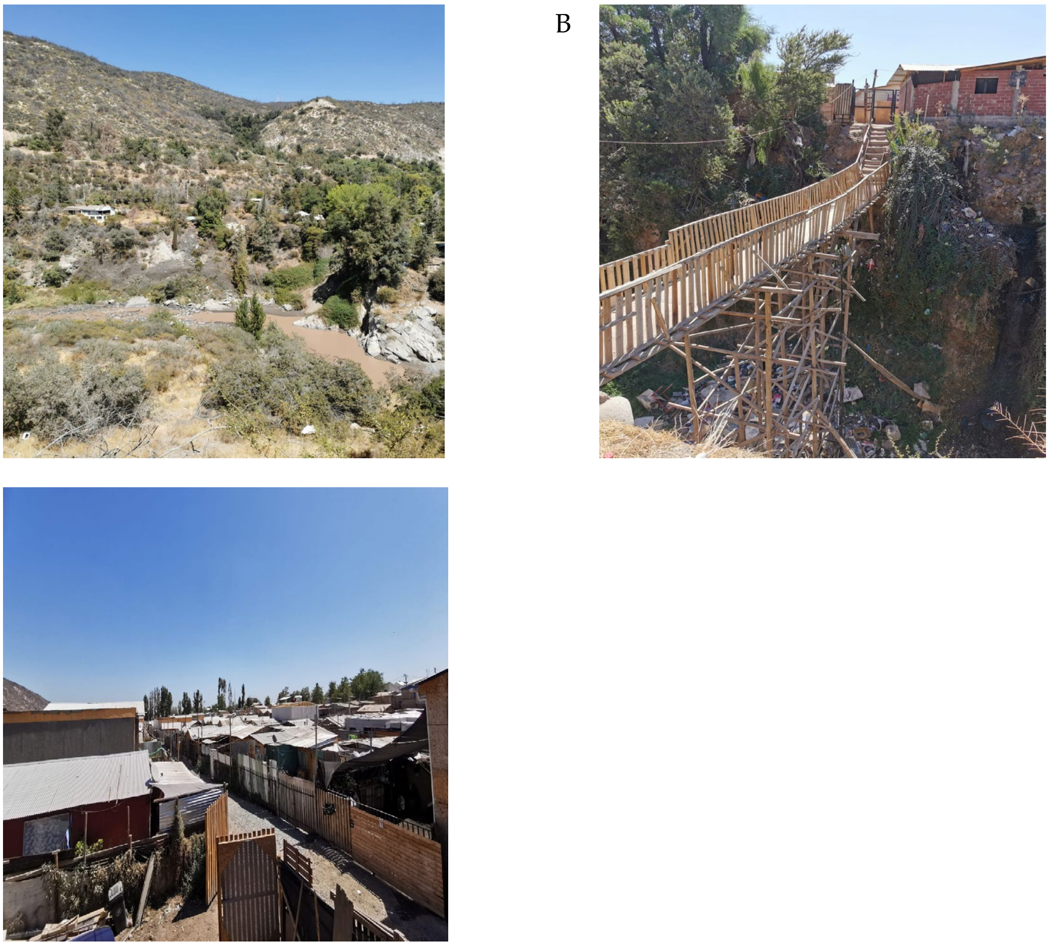

Figure S7. Informal settlement located near the study area. (A) Peripheral location of the settlement in relation to the urban area; (B) lack of adequate access infrastructure; (C) high density of dwellings concentrated in a reduced space.

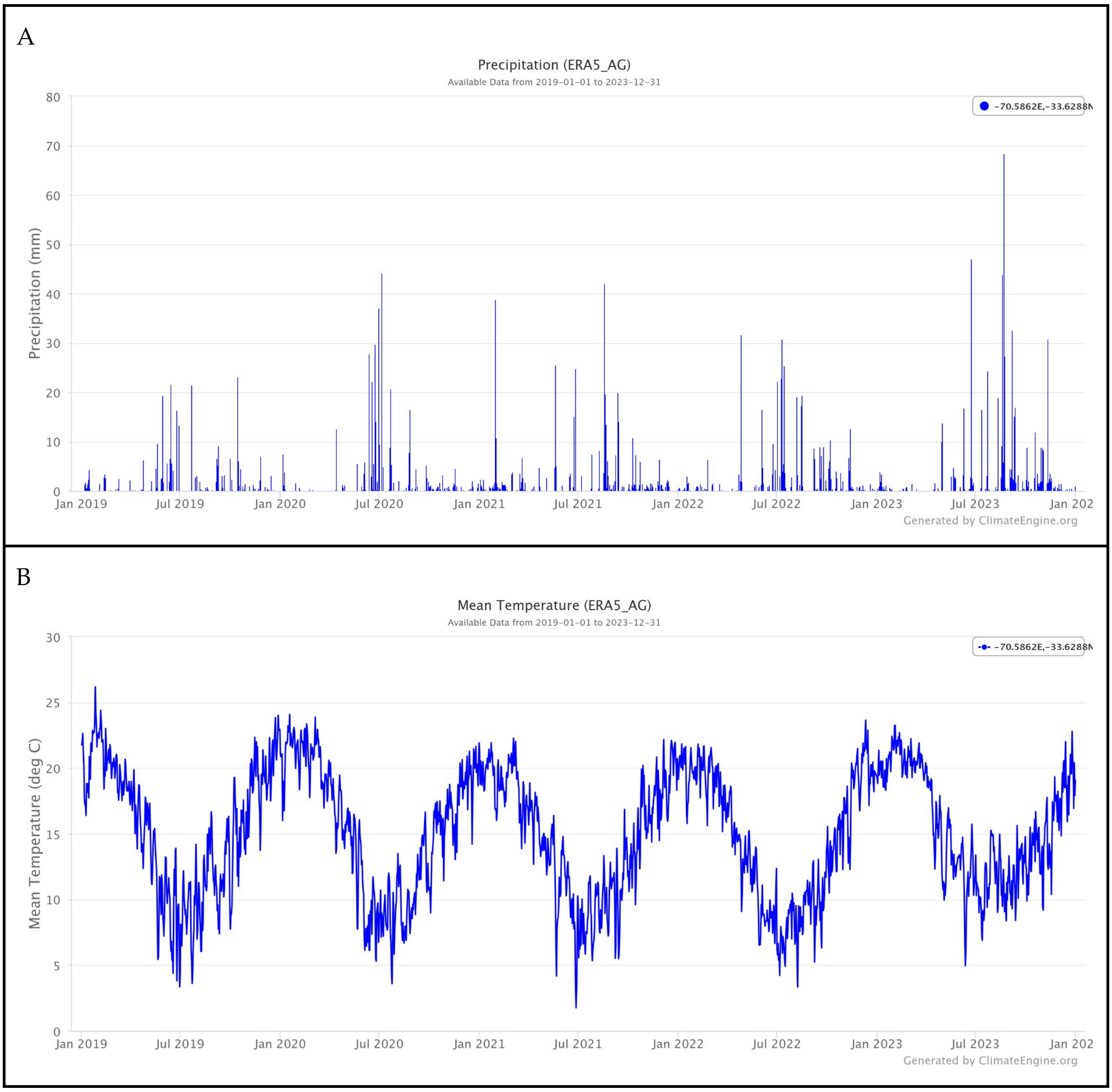

Figure S8. Temporal series of precipitation (A) and mean temperature (B) of a point (-70.58E ; -33.62N) in the surroundings of the Maipo River.

Table S2: Equations for Index

NDVI (Normalized Difference Vegetation Index)
NDVI = (NIR - Red) / (NIR + Red)
Using Sentinel-2 Bands: NDVI = (Banda 8 - Banda 4) / (Banda 8 + Banda 4)

SAVI (Soil Adjusted Vegetation Index)
SAVI = [(NIR - Red) * (1 + L)] / (NIR + Red + L) Where L is the soil adjustment factor (commonly 0.5).
Using Sentinel-2 Bands: SAVI = [(Banda 8 - Banda 4) * (1 + L)] / (Banda 8 + Banda 4 + L)

EVI (Enhanced Vegetation Index)
EVI = 2.5 * [(NIR - Red) / (NIR + 6 * Red - 7.5 * Blue + 1)]
Using Sentinel-2 Bands: EVI = 2.5 * [(Banda 8 - Banda 4) / (Banda 8 + 6 * Banda 4 - 7.5 * Banda 2 + 1)]

GCI (Green Chlorophyll Index)
GCI = (NIR / Green) - 1
Using Sentinel-2 Bands: GCI = (Banda 8 / Banda 3) - 1

GNDVI (Green Normalized Difference Vegetation Index)
GNDVI = (NIR - Green) / (NIR + Green)
Using Sentinel-2 Bands: GNDVI = (Banda 8 - Banda 3) / (Banda 8 + Banda 3)

NDMI (Normalized Difference Moisture Index)
Also known as NDWI (Gao). NDMI = (NIR - SWIR1) / (NIR + SWIR1)
Using Sentinel-2 Bands: NDMI = (Banda 8 - Banda 11) / (Banda 8 + Banda 11)

NDWI (Normalized Difference Water Index)
Refers to the McFeeters NDWI. NDWI = (Green - NIR) / (Green + NIR)
Using Sentinel-2 Bands: NDWI = (Banda 3 - Banda 8) / (Banda 3 + Banda 8)

MNDWI (Modified Normalized Difference Water Index)
MNDWI = (Green - SWIR1) / (Green + SWIR1)
Using Sentinel-2 Bands: MNDWI = (Banda 3 - Banda 11) / (Banda 3 + Banda 11)

AWEI (Automated Water Extraction Index)
Assuming AWEI_shadow (AWEI_sh). AWEI_sh = Blue + 2.5 * Green - 1.5 * (NIR + SWIR1) - 0.25 * SWIR2
Using Sentinel-2 Bands: AWEI_sh = Banda 2 + 2.5 * Banda 3 - 1.5 * (Banda 8 + Banda 11) - 0.25 * Banda 12

LSWI (Land Surface Water Index)
LSWI = (NIR - SWIR1) / (NIR + SWIR1)
Using Sentinel-2 Bands: LSWI = (Banda 8 - Banda 11) / (Banda 8 + Banda 11)

NDBI (Normalized Difference Built-up Index)
NDBI = (SWIR1 - NIR) / (SWIR1 + NIR)
Using Sentinel-2 Bands: NDBI = (Banda 11 - Banda 8) / (Banda 11 + Banda 8)

NDTI (Normalized Difference Tillage Index)
Commonly uses SWIR bands for residue detection. NDTI = (SWIR1 - SWIR2) / (SWIR1 + SWIR2)
Using Sentinel-2 Bands: NDTI = (Banda 11 - Banda 12) / (Banda 11 + Banda 12)
